# Construction of a prognostic survival model for colorectal cancer patients using CT image texture analysis: a prospective cohort study

**DOI:** 10.3389/fonc.2025.1738696

**Published:** 2026-01-12

**Authors:** Chen-hua Sun, Hao-di Wang, Wen-hao Sun, Guan-wen Gong, Zheng-ming Deng, Zhi-wei Jiang

**Affiliations:** 1 Affiliated Hospital of Nanjing University of Chinese Medicine, Nanjing, Jiangsu, China; 2 Jiangsu Province Hospital of Chinese Medicine, Nanjing, Jiangsu, China; 3 Nanjing University of Chinese Medicine, Nanjing, Jiangsu, China

**Keywords:** colorectal cancer, LASSO regression, nomogram, survival prediction model, texture analysis

## Abstract

**Background:**

Current prognostic indicators for colorectal cancer are limited to pathological staging, which offer only modest predictive value. This study aims to develop a prognostic prediction model for colorectal cancer patients based on texture analysis (TA), with the goal of forecasting long-term survival outcomes.

**Methods:**

A total of 236 patients underwent abdominal CT scanning, including both unenhanced and contrast-enhanced CT. Using MaZda software, regions of interest (ROIs) were identified, and texture features were extracted. These texture features were combined with pathological staging data, and statistical analyses were performed using Cox regression, Lasso regression, nomograms, forest plots, receiver operating characteristic (ROC) curve analysis, and survival analysis (Kaplan-Meier curves), and carry out the validation work of the external validation set.

**Results:**

Observation points were established at 1, 3 and 5 years. A correlation analysis was conducted using patient demographic data, tumor markers, pathological staging, and more than 300 variables derived from the texture analysis. The analysis revealed correlations between texture features (such as Teta1, Teta4, WavEnLL_s-2, GrSkewness, and Horzl_RLNonUni) and survival time. Nomograms were created to provide a rough estimation of patient survival, which could assist in decision-making for subsequent treatment plans. Using Lasso regression combined with the nomogram for dimensionality reduction, we were able to intuitively assess the predicted five-year survival time for patients in the perioperative period.

**Conclusion:**

Radiomics analysis of colorectal cancer, when combined with traditional TNM staging, can aid in the construction of a survival prediction model. This model may offer novel insights for predicting long-term survival and provide a reference for the development of individualized treatment strategies.

**Clinical trial registration:**

## Highlights

- The progression of colorectal cancer at various stages can be clearly reflected in imaging data, such as CT scans.
- Traditional methods for predicting the prognosis of colorectal cancer patients heavily rely on postoperative TNM staging, often overlooking valuable preoperative imaging information.
- By integrating quantitative imaging data with conventional evaluation metrics, more precise prognostic prediction models can be developed.
- These advanced models offer targeted guidance for formulating comprehensive and personalized treatment strategies for colorectal cancer.

## Introduction

Colorectal cancer is currently the third most common malignant tumor worldwide and the second leading cause of cancer-related death globally (1). The high mortality rate among colorectal cancer survivors can be attributed to disease recurrence, with an estimated 29%–63% of patients diagnosed with locally advanced disease experiencing relapse. The TNM staging system, based on tumor infiltration depth (T), lymph node metastasis (N), and distant metastasis (M), is closely associated with the five-year survival rate of colorectal cancer patients (2). However, apart from TNM staging, there are few robust predictive indicators for long-term survival.

Although screening methods and treatment strategies have advanced in recent years, research on cancer prevention and therapeutic biomarkers has never ceased. Well-known mutations in colorectal cancer cells include KRAS, TP53, and others (3, 4). While these studies provide some guidance for colorectal cancer diagnosis and intervention (5), there remains a lack of convincing quantitative markers for prognostic prediction, aside from the TNM staging system.

The growth of colorectal cancer is accompanied by the activation of numerous biological processes, such as angiogenesis, altered cellular metabolism, and increased glucose consumption. These processes contribute to tumor heterogeneity, characterized by abnormal, irregular, and disordered tissue structures, along with high cellular density, hypoxia, necrosis, hemorrhage, and mucinous changes. Tumor heterogeneity tends to evolve over time, increasing with tumor progression and affecting local and distant invasion, chemotherapy delivery, and cellular resistance to chemotherapy and radiotherapy, ultimately impacting prognosis (6). Given this complex disease progression, our goal is to identify subtle clues amidst this chaos, enabling early diagnosis and intervention to improve patient outcomes.

For patients with locally advanced colorectal cancer, accurately predicting long-term survival during the perioperative period could provide crucial insights for individualized treatment planning, including neoadjuvant and adjuvant chemoradiotherapy. This approach could significantly reduce recurrence rates and mortality (7). Currently, advanced imaging technologies such as CT, MRI, and PET-CT offer opportunities to obtain structural, functional, and molecular information about tumors (8). Among these, CT remains the most time-efficient and cost-effective modality. However, CT image interpretation primarily relies on human expertise, which can be prone to errors, particularly in cases where the radiologist lacks experience. Even experienced radiologists may disagree on complex cases, primarily due to the inherent subjectivity in CT image interpretation. Moreover, traditional quantitative indicators from CT, such as Hounsfield units, have limited predictive power regarding patient prognosis, and research into prognostic prediction remains sparse.

In recent years, with the rapid advancement of artificial intelligence (AI), there have been profound changes in various sectors, including healthcare. AI has also found applications in medical imaging, particularly in texture analysis (TA), a radiomics technique that quantifies medical images. TA enables a detailed assessment of the heterogeneity of lesions and surrounding tissues, addressing the limitations of human vision, and offering precise, quantitative data extraction from medical images. TA has been widely applied in the preoperative diagnosis, pathological grading, and prognostic prediction of pancreatic cancer (9, 10). Texture analysis is based on traditional medical imaging, and it quantifies tumor heterogeneity by analyzing the distribution and relationships of pixel or voxel grayscale values in the lesion and surrounding tissues (11, 12). As a non-invasive imaging tool, TA has shown great potential in the diagnosis, treatment assessment, and prognosis of rectal cancer (13).

In this study, our team collected texture analysis data from colorectal cancer patients who underwent radical surgery at our center in 2019. By integrating this data with general clinical information and TNM staging, as well as the five-year survival data of the patients, we aim to develop a prognostic model for estimating the survival time of colorectal cancer patients. Utilizing machine learning-based dimensionality reduction techniques and visual tools such as nomograms, our goal is to provide a more robust foundation for personalized diagnosis and treatment. Our article is compliant with the TITAN Guidelines 2025 (14).

## Materials and methods

### Patient selection

We conducted an analysis of records from patients who underwent full-abdominal enhanced CT scans, which showed significant enhancement of lesions in part of the intestinal wall, between January 2019 and December 2019. The exclusion criteria were as follows: 1) patients who received anti-tumor therapy prior to the CT examination; 2) patients with poor image quality that hindered lesion segmentation; 3) Patients with confirmed liver metastasis before surgery were temporarily excluded from this study and will be included in future analyses once enough cases are available; 4) Patients were followed up for a period of 5 years, and cases with missing follow-up data were excluded from the study. All patients underwent primary radical colon surgery, and the postoperative chemotherapy regimen followed the standard XELOX protocol. Patients without tumor progression were scheduled for regular follow-up examinations, while those with tumor progression were treated with the standard combination therapy of bevacizumab for both left-sided and right-sided colon tumors.

This study has been registered with the Chinese Clinical Trial Registry. Initially, 296 patients were enrolled in the study. Among them, 6 patients presented with distant metastases (e.g., hepatic metastasis) at initial diagnosis, and 20 patients opted for preoperative neoadjuvant therapy. All participants underwent a 5-year survival follow-up, during which 34 cases were lost to follow-up. Ultimately, 236 patients were included in the final analysis. Furthermore, in collaboration with three partner hospitals, our research team collected external validation cohort data using the aforementioned inclusion and exclusion criteria. After excluding patients who received neoadjuvant therapy or had distant metastases to organs such as the liver, we enrolled cases with complete 5-year follow-up information. The three institutions contributed 118, 62, and 20 cases respectively. The work has been reported in line with the STROCSS criteria (15). The negative control group is the group of benign tumors, classified as Group 0 in the raw data. The follow-up period in our study was truncated at 60 months.

### Image selection and classification based on texture analysis

**Figure 1:**
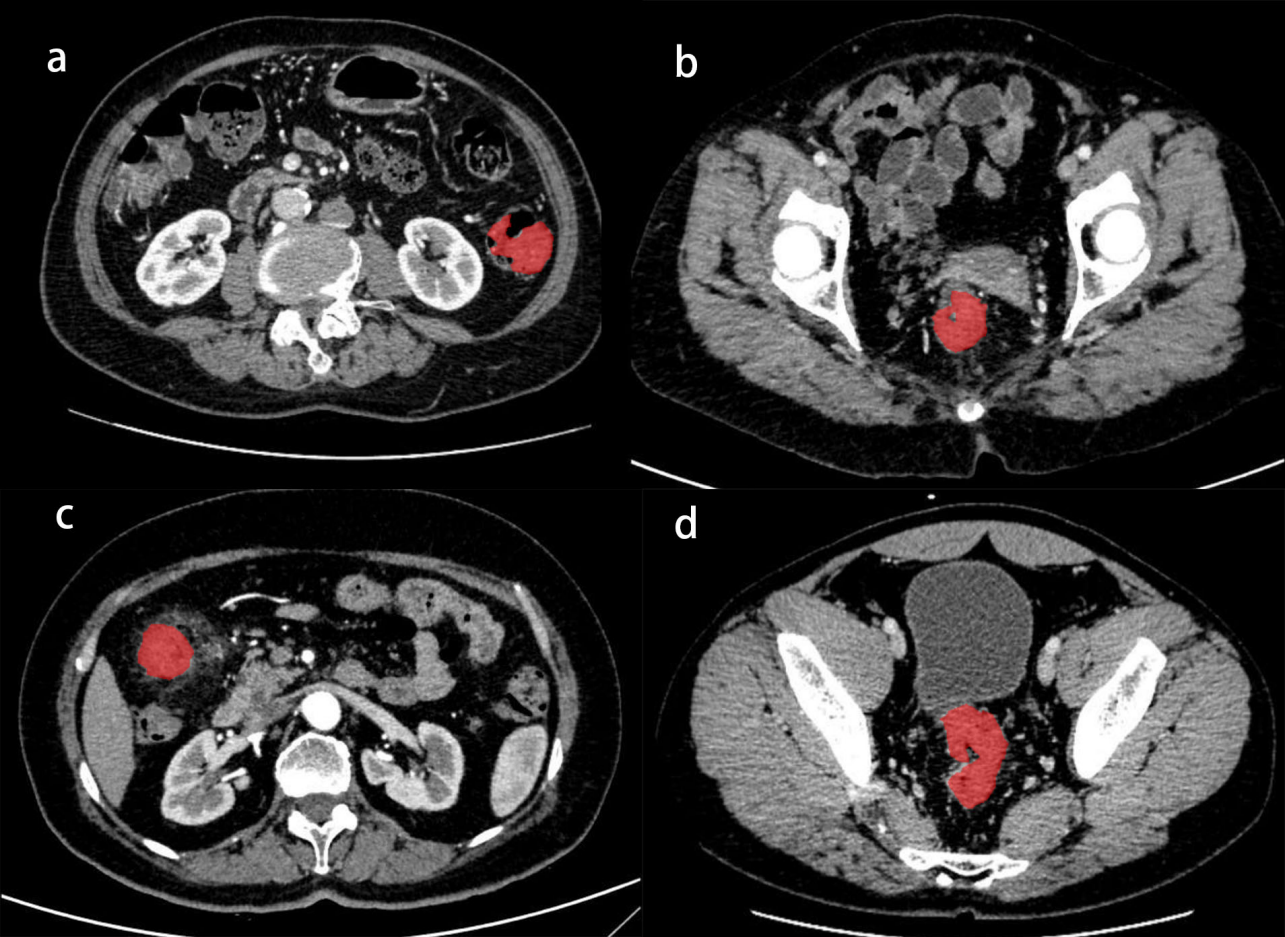
The selection of ROI of CT images using MaZda software. **(A)** ROI of the mass in the descending colon; **(B)** ROI of the mass in the rectum; **(C)** ROI of the mass in the ascending colon; **(D)** ROI of the mass in the sigmoid colon.

The criteria for image selection were as follows: (A) colonic wall thickening or lesion thickness greater than 5mm; (B) visible significant enhancement of the intestinal wall in the image; (C) preference for asymmetric or localized colonic thickening. CT images were reviewed, and the largest tumor cross-section was selected by two gastrointestinal radiologists on three-dimensional (3D) images. The regions of interest (ROIs) were delineated using MaZda software. ROIs selection was independently performed by two chief radiologists, each with over 10 years of clinical experience. In cases where significant discrepancies existed between their selections, a third senior radiologist (similarly qualified with ≥10 years’ experience) was consulted to reach consensus, thereby finalizing the radiomics data acquisition process. Discrepancies between radiologists were resolved through consensus. The selected ROIs encompassed the entire tumor, avoiding vascular structures, calcification, and gas (**Figure 1**). A total of 302 texture-based quantitative features were automatically extracted from the ROIs for inclusion in the study.

### Statistical analysis

**Figure 2:**
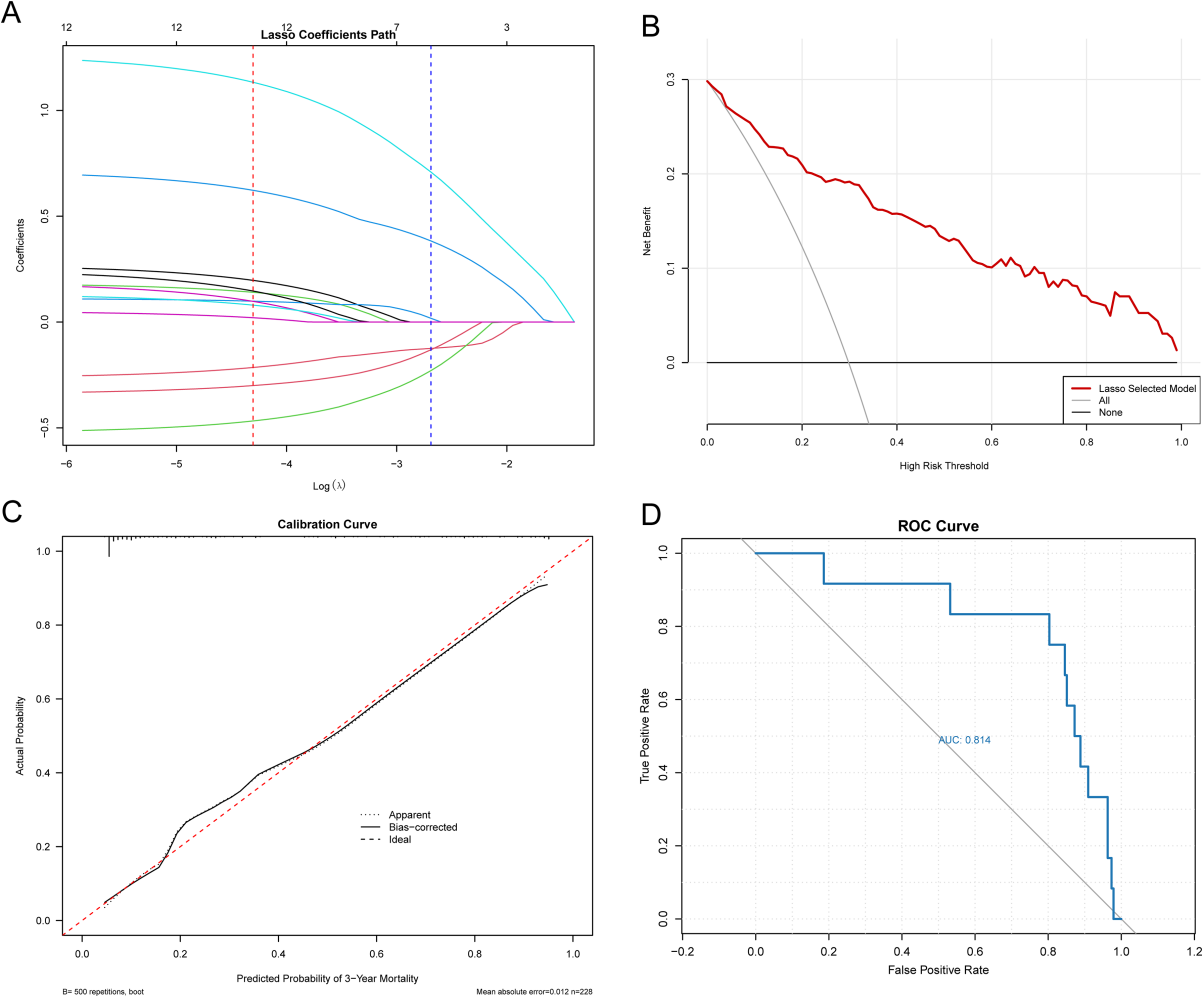
Evaluation of the nomogram survival prediction model. **(A)** Coefficient path plots were generated with the optimal lambda value indicated as a reference line; **(B)** Evaluating the Predictive Performance of the LASSO Model Using Decision Curve Analysis (DCA); **(C)** Utilizing calibration curves to verify the accuracy of predictive models; **(D)** Receiver Operating Characteristic (ROC) Curve of the External Validation Cohort.

Statistical analysis was performed using R 4.4.2. This study employed analytical methodologies including univariate and multivariate Cox regression, LASSO regression, nomograms, and forest plots. LASSO regression analysis employed 10-fold cross-validation to determine the optimal lambda value (i.e., the regularization parameter in LASSO regression), which was subsequently used to fit the final model. The cross-validation curve of LASSO regression was plotted to visualize the model selection process. Additionally, a coefficient path plot (**Figure 2a**) was generated with the optimal lambda value marked as a reference line. For variables selected by LASSO regression, further analyses were performed, including the construction of a forest plot to display effect sizes and a nomogram for clinical prediction. To validate the predictive performance of the model, decision curve analysis (DCA) and calibration curves were also completed. And receiver operating characteristic (16) curve analysis were conducted using the 302 quantitative features derived from texture analysis, in combination with patient demographic data, including age, sex, alcohol consumption, CEA, CA199 levels, and TNM staging. The TNM staging system used in this study was based on the 8th edition of the AJCC (American Joint Committee on Cancer) staging criteria for colorectal cancer. This standardized staging system was consistently applied throughout both the data collection and analytical phases of the research. Data dimensionality reduction and other machine learning-based data processing methods were applied. Correlation analyses were conducted using perioperative data and patient survival information to develop a prognostic prediction model for colorectal cancer survival. Additionally, an external validation cohort comprising 200 cases was independently assessed using receiver operating characteristic (ROC) curve analysis to evaluate the predictive performance of the model. To enable a more precise quantitative comparison between different predictive models, we further compared the model incorporating radiomics features with the model using TNM staging alone by calculating the integrated discrimination improvement (IDI) and net reclassification improvement (NRI).

## Results

**Figure 3:**
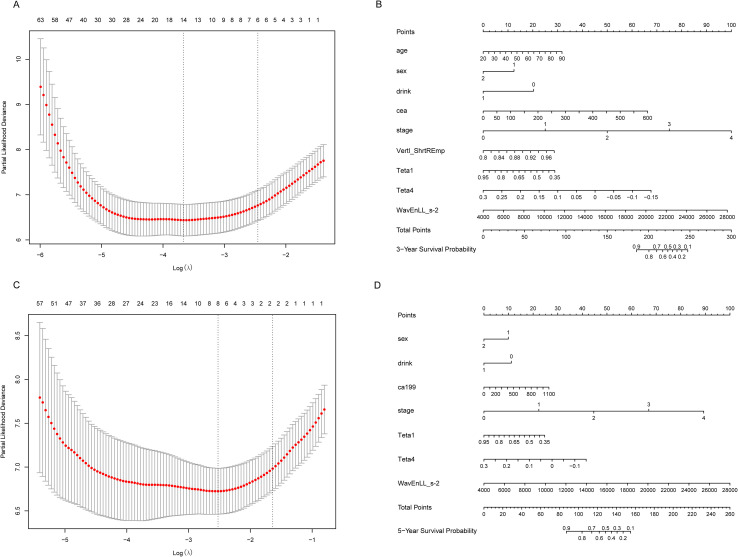
LASSO regression and nomograms for 3 and 5 years. **(A, C)** Select the most critical variables for predicting 3-year and 5-year survival rates with the optimal Log(λ); **(B, D)** Construct nomograms for 3-year and 5-year survival rates based on the selected predictor variables.

Firstly, the MaZda software was used to delineate the regions of interest (ROIs) from the CT images of 236 patients, from which over 300 texture analysis quantitative features, including Teta1, Teta4, WavEnLL_s-2 and GrSkewness were extracted. These 302 variables, combined with patient data such as age, sex, alcohol consumption, CEA, CA199 levels, and TNM staging, were analyzed in correlation with patient survival data. The survival status at 1, 3, and 5 years was used as the outcome variable, with survival or death as the gold standard. LASSO regression and nomograms were employed to perform dimensionality reduction and visualize predictive models for 1-year, 3-year, and 5-year survival, and we performed 10-fold cross-validation to determine the optimal lambda value (the regularization parameter in LASSO regression), which was used to fit the final LASSO model. The cross-validation curves of LASSO regression were plotted (**Figures 3a, c**, and **Supplementary Figure S1a**). Additionally, coefficient path plots were generated with the optimal lambda value indicated as a reference line (**Figure 2a**). For the 1-year data, the optimal lambda value was 0.0296. The variables selected included CA199, CEA, stage, Horzl_RLNonUni, GrSkewness, and WavEnLL_s-5. After multivariate stepwise regression, the corresponding nomogram was generated (**Supplementary Figure S1b**). Similarly, for the 3-year and 5-year data, the optimal lambda values were 0.0256 and 0.0802, respectively, and after stepwise regression, their respective nomogram prediction models were generated (**Figures 3b, d**).

It is evident from the analysis that, in addition to common preoperative data such as tumor markers (e.g., CA199, CEA), and general patient information (e.g., age, sex), as well as TNM staging data, the CT texture analysis variables showing significant predictive value include Teta1, Teta4, WavEnLL_s-2, GrSkewness, Horzl_RLNonUni, and WavEnLL_s-5. Among these, Teta1, Teta4, and WavEnLL_s-2 demonstrated strong prognostic significance in the 3-year and 5-year prediction models (**Table 1**), while Horzl_RLNonUni, GrSkewness, and WavEnLL_s-5 showed notable predictive value for the 1-year model (**Table 1**). Furthermore, the forest plot analysis revealed statistically significant results for Teta1, Teta4, and WavEnLL_s-2 (**Supplementary Figure S2**).

**Table 1:** Comparison of texture feature among colonic enhanced imaging in different survival data.

Group	TA feature	Hazard Ratio	95% CIs	*P* value
1Y	Horzl_RLNonUni	1.0005	(1.00-1.20)	0.027
	GrSkewness	2.8446	(1.10-6.70)	0.002
	WavEnLL_s-5	1.0001	(1.00-1.00)	0.782
	TNM	2.7108	(1.00-7.20)	0.049
3Y	Vertl_ShrtREmp	0.6100	(0.02-2.90)	0.070
	Teta1	0.1063	(0.01-1.10)	0.062
	Teta4	0.0150	(0.0021-0.12)	0.001
	WavEnLL_s-2	1.0002	(1.00-1.20)	0.001
	TNM	3.3769	(2.40-4.80)	0.001
5Y	Teta1	0.1537	(0.02-1.15)	0.068
	Teta4	0.0149	(0.00-0.27)	0.005
	WavEnLL_s-2	1.0002	(1.00-1.00)	0.001
	TNM	2.7648	(2.19-3.50)	0.001

**Figure 4:**
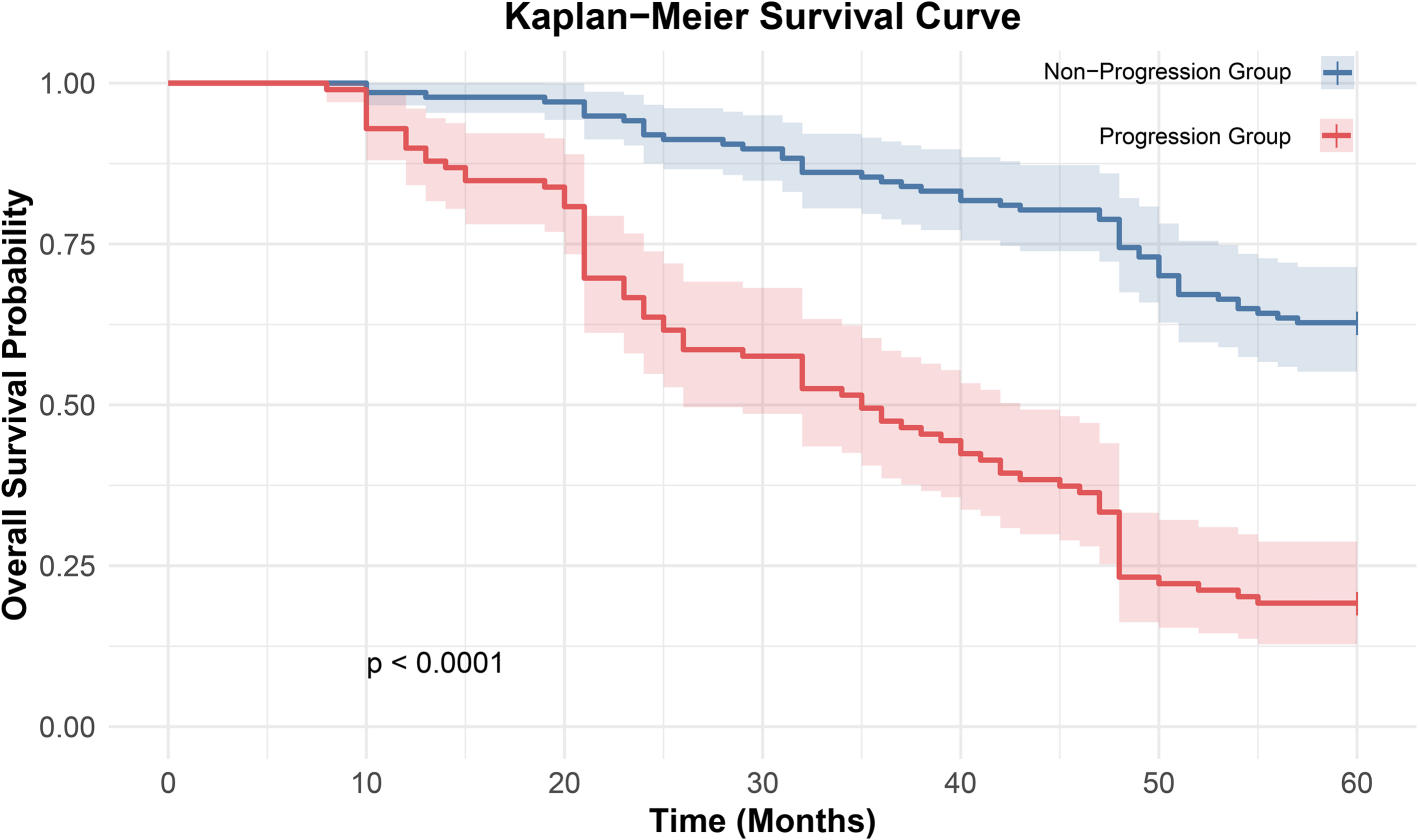
Kaplan-Meier survival curves for progression and non-progression patients.

**Figure 5:**
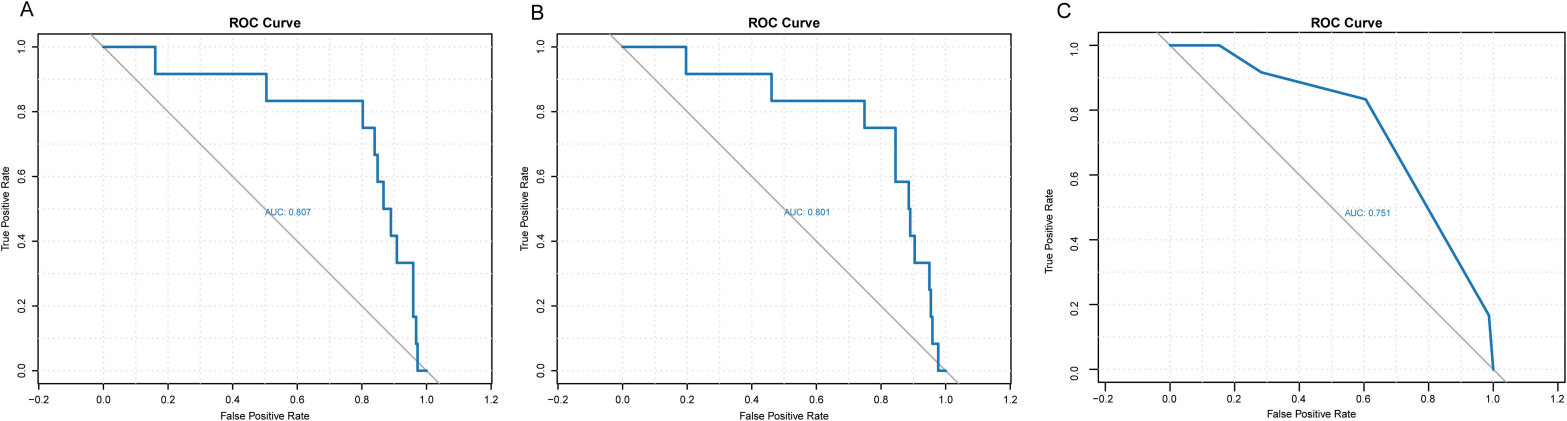
ROC curves of texture analysis results and general patient data. **(A)** The Receiver Operating Characteristic (ROC) Curve of CT texture analysis variables combined with TNM staging data; **(B)** The ROC curve of CT texture analysis variables; **(B)** The ROC curve of TNM staging data.

Additionally, we found that TNM staging inherently holds predictive advantages for prognosis (**Table 1**). The results clearly indicate that patients with advanced-stage colorectal cancer have a significantly shorter expected survival time compared to those with non-advanced disease (**Figure 4**). However, its ability to predict specific survival times remains limited. In the ROC curve analysis, we observed that the AUC for CT texture analysis variables was 0.801, while the AUC for TNM staging data was 0.751. When CT texture analysis and TNM staging were combined, the overall AUC increased to 0.807 (**Figure 5**). Finally, we incorporated both radiomics features and TNM staging alone as variables in the predictive models. To quantitatively compare the performance improvement between models with different variable combinations, our research team employed Integrated Discrimination Improvement (IDI) and Net Reclassification Improvement (NRI) for further evaluation. We calculated net reclassification improvement (NRI=0.460, p<0.01) and integrated discrimination improvement (IDI=0.217, p<0.05), demonstrating significant reclassification improvement. This indicates that, in combination with traditional TNM staging, CT texture analysis significantly supplements aspects not covered by TNM staging, offering a more comprehensive prediction model for patient prognosis. Furthermore, we performed a survival curve analysis on the 236 enrolled colorectal cancer patients to visually evaluate their five-year survival outcomes.

To further evaluate the predictive performance of the LASSO-derived radiomics model, we constructed decision curves and calibration curves. As shown in **Figure 2b**, the LASSO model demonstrated significant clinical utility, particularly at intermediate-to-low risk thresholds, where its decision-making efficacy was most pronounced. **Figure 2c** illustrates the calibration curve after 500 bootstrap iterations, revealing near-perfect alignment between predicted and observed mortality probabilities, indicating excellent calibration and high reliability. Furthermore, external validation with an independent cohort of 200 cases yielded an AUC of 0.814 (**Figure 2d**), which showed no significant difference compared to the original training cohort AUC of 0.807, confirming the robust predictive performance of our model.

## Discussion

The purpose of this study was to incorporate key imaging information, which is considered crucial in clinical practice, into the research framework and develop a novel predictive approach. By utilizing real-world data from a five-year follow-up at our center, we aimed to construct a prediction model. As described earlier, using MaZda software, 302 quantitative texture features of tumor tissue were derived and combined with patient demographics, along with survival data over five years. Univariate and multivariate Cox regression analyses, LASSO regression, nomogram analysis, forest plots, and ROC curve analysis were performed. Through dimensionality reduction with the machine learning techniques mentioned above and deep integration with traditional TNM staging data, we were able to more accurately predict the approximate survival status of colorectal cancer (CRC) patients over the next five years during the perioperative period, providing strong support for clinical decision-making.

Reviewing traditional imaging diagnostic experience, experienced experts typically rely on the degree of tumor enhancement and the presence of significantly enlarged lymph nodes around the tumor to guide diagnosis. More specifically, lymph node metastasis is tracked to different stations. Traditional quantification tools, such as CT values, are commonly used. Tumor markers such as CEA and CA199, which are recognized to have certain guiding significance for tumor progression, also serve as useful references for assessing the severity of tumors. However, traditional analysis methods often fail to provide homogeneous conclusions about tumor malignancy, progression, and expected survival time, heavily depending on the clinical experience of the physician. With the addition of traditional TNM staging information, clinicians can make a general prognosis based on serum markers and basic patient information, such as age, sex, smoking, drinking habits, and BMI. Yet, preoperative CT imaging, which is crucial for assessing tumor prognosis, lacks further quantifiable data to support such predictions, which is a significant limitation.

In recent years, with the rapid development of artificial intelligence (AI), numerous imaging radiomics studies have been conducted on CT imaging in CRC. We are now shifting focus not only on qualitative analysis of images but also on accurate quantitative diagnostic work. Texture analysis (TA) has been analyzed for its potential and feasibility in assisting precise diagnosis (8). Recent literature has increasingly highlighted the utility of CT as a non-invasive diagnostic tool. It has been found that CT can distinguish between benign and malignant colorectal tumors in asymptomatic patients (17, 18). Volumetric analysis suggests that for tumors larger than 3 cm, texture analysis can nearly match human experience in distinguishing benign from malignant tumors, although it remains complementary. Studies have shown that TA significantly outperforms resident physicians in classifying CRC, inflammatory bowel disease (IBD), and normal thickening of the colon (NTC), though there is no significant difference when compared with experienced radiologists (19). In the early stages of this study, we also performed a series of validation tests on this result, confirming that CT radiomics data can assist in preoperative assessment of tumor benignancy and approximate malignancy (**Table 2**). Furthermore, image-based radiomics using pre-processed CT data has been shown to predict the outcomes of hepatic arterial infusion chemotherapy (HAIC) for advanced inoperable CRC with high accuracy and feasibility (13). Texture analysis data based on MRI and PET-CT can assist in predicting CRC recurrence risks and evaluating the effects of radiotherapy (20, 21).

**Table 2:** Comparison of texture feature among colonic enhanced imaging in different pathological stages.

Group	TA feature	Mean ± Std. Dev.	t	*P* value
Negative control	Area	2768 ± 1533	Base outcome	
	Mean	109 ± 16		
	Variance	1059 ± 480		
	Skewness	-0.91 ± 0.60		
	Kurtosis	1.86 ± 2.77		
I	Area	2360 ± 706	-2.63	**0.009**
	Mean	129 ± 10	3.99	**0.000**
	Variance	1145 ± 525	0.07	0.946
	Skewness	-0.82 ± 0.23	1.19	0.235
	Kurtosis	1.40 ± 1.07	-0.58	0.561
II	Area	4558 ± 2399	1.58	0.115
	Mean	124 ± 10	2,77	**0.006**
	Variance	1058 ± 393	-0.87	0.386
	Skewness	-0.89 ± 0.34	-0.61	0.544
	Kurtosis	1.23 ± 1.00	-2.32	**0.020**
III	Area	4573 ± 1606	1.40	0.162
	Mean	128 ± 11	3.54	**0.000**
	Variance	1069 ± 374	-0.74	0.460
	Skewness	-0.89 ± 0.35	-0.25	0.805
	Kurtosis	1.32 ± 1.09	-2.25	**0.024**

Bold font indicates statistical significance.

However, there are few reports on studies analyzing the correlation between CT-based radiomics features and patient survival prognosis, as well as the construction of predictive models. Instead, most research has focused on utilizing MRI and PET-CT imaging data, which exhibit relative disadvantages in terms of clinical generalizability and convenience compared to CT examinations. Abdominal CT is relatively low-cost and enjoys higher public acceptance. Moreover, during the initial diagnostic process, clinicians are more inclined to prescribe abdominal CT examinations. Additionally, for the establishment of prognostic models, this study investigates the correlation between radiomics features and patient survival time, thereby constructing 1-year, 3-year, and 5-year predicted prognostic model. Compared to the traditional TNM staging system, radiomics features demonstrate superior predictive capability for prognosis. This approach allows for a preoperative estimation of patient survival time, providing an additional dimension of evidence to support personalized clinical decision-making regarding treatment strategies.

For patients with CRC liver metastases, TA can predict the efficacy of different chemotherapy regimens (22). For CRC patients without distant metastases, image-based global tumor features have been shown to predict pathological staging of rectal cancer preoperatively, thus providing a theoretical basis for individualized treatment, adjuvant chemotherapy, and even neoadjuvant chemotherapy (23).

Texture analysis also demonstrates diagnostic advantages for diseases in other systems. In the context of the global COVID-19 pandemic, current diagnostic guidelines recommend RT-PCR testing. As an auxiliary diagnostic tool, chest CT has been shown to reveal visual features of COVID-19 and provides clear guidance for several stages of the disease. A semi-supervised learning framework for 3D segmentation of COVID-19 infection areas has been proposed from chest CT scans to achieve accurate diagnosis and treatment (24). Texture analysis can also play a guiding role in clinical staging prediction and recurrence risk assessment of lung cancer, as well as in diagnosing and differentiating pancreatic neuroendocrine tumors based on enhanced CT radiomics features (25–27). Additionally, for diseases that are difficult to biopsy preoperatively, texture analysis has proven to be a strong aid in the prognosis and diagnosis of glioblastoma and brain metastases (28, 29).

In recent years, especially in the last two years, the rapid advancements in artificial intelligence and computer technology have shown clear advantages over humans in many fields (30). This study, by utilizing a visualized prognosis prediction model, provides a direct way to assess the expected five-year survival status of CRC patients during the perioperative period. For CRC patients, after fully assessing their cardiopulmonary function, first-stage R0 radical resection of the lesion has become the clinical treatment of choice. The postoperative management of comprehensive treatment may vary across centers. This study retrospectively collected CRC surgical cases from our center in 2019. By strict inclusion and exclusion criteria, patients who underwent neoadjuvant therapy or had preoperative liver metastasis were excluded, and only patients who underwent first-stage surgery with XELOX chemotherapy were included.

By integrating radiomics data with traditional patient demographics and TNM staging data, and applying dimensionality reduction and machine learning methods, this study provides a visualized model for predicting CRC patient survival within five years. Notably, we discovered that the predictive efficacy of pure radiomics data was not inferior to that of TNM staging alone, and the combination of both resulted in an even better prognosis prediction model. This offers a more comprehensive prediction of patient survival over the next 1, 3, or even 5 years. Thus, our study fills the gap where preoperative CT lacks quantitative guidance for assessing CRC patient prognosis, providing multidimensional data support for personalized treatment adjustments.

The prognostic improvement of the predictive model proposed in this study, compared to the conventional TNM staging system, is primarily reflected in the following aspects: First, based on data from our center and external validation cohorts, repeated analyses demonstrated that the AUC values of radiomics feature-based indicators showed no statistically significant difference from those of the TNM staging system alone, and even slightly outperformed the latter. This indicates that the proposed model, utilizing quantitative imaging biomarkers, can provide independent prognostic predictive capability. Second, compared to the inherent delay in acquiring traditional TNM staging data—which requires postoperative pathological examination after radical surgery to obtain a complete and relatively accurate staging—radiomics data can be accurately obtained prior to surgical intervention. This allows for earlier estimation of disease stage, offering strong clinical guidance for personalized treatment planning, particularly in low-income populations from underdeveloped regions, where decisions regarding aggressive systemic therapies must be carefully weighed. Finally, accurate TNM staging strictly depends on patients undergoing standard radical surgical procedures, as only then can a reliable pathological stage be determined. However, in real-world practice, the surgeon’s technical proficiency may limit the extent of lymph node dissection, potentially compromising the radicality of resection, which critically impacts pathological staging accuracy. In contrast, radiomics data can be objectively and reproducibly acquired preoperatively, thereby providing a novel complementary dimension to the conventional pathological staging system.

The prognostic prediction model in this study was constructed through a multidimensional approach integrating radiomic features, conventional TNM staging data, and relevant serological biomarkers, with patients’ actual survival time serving as the study endpoint. In collaboration with three hospitals, our research team collected external validation cohort data and performed corresponding external validation. The model demonstrated an AUC of 0.814, showing no significant deviation from the original study data (p>0.05), thereby confirming its robust predictive performance. Notably, as previously reported, the radiomic signature alone achieved an AUC of 0.807, which was not inferior to the AUC of 0.751 obtained using TNM staging alone (NRI=0.460, p<0.01; IDI=0.217, p<0.05). In the external validation cohort, TNM staging alone yielded an AUC of 0.750. These results collectively demonstrate that our model provides a universally applicable tool for prognostic prediction in colorectal cancer patients.

However, there are limitations to this study. Texture analysis relies on accurate annotation of the regions of interest (ROIs), which requires experienced clinicians or senior radiologists, and cannot yet fully automate prognosis prediction. In the actual practice of selecting accurate ROIs (Regions of Interest), significant subjective factors remain. To address this, our research team employed two board-certified radiologists, each with over ten years of experience, to independently delineate the ROIs. In cases of substantial disagreement, a third senior radiologist (similarly qualified with ≥10 years’ experience) was consulted for arbitration. This triple-validation protocol effectively minimizes measurement variability attributable to manual segmentation. Furthermore, given that the sample size in this study is not large, further validation with larger sample sizes is needed, especially in patients with confirmed preoperative liver metastasis. After expanding the number of cases, further studies should be conducted. If possible, multi-center cohort studies could be initiated to further refine the CRC prognosis prediction model and provide stronger support for individualized treatment. By using the nomogram model to predict the 1-year, 3-year and 5-year survival rates of patients, we can estimate patients’ expected lifespan more intuitively. Although this decision is quite difficult, it provides more quantitative numerical references for the choice between active clinical treatment and conservative palliative treatment. As for the future application scenarios and practical application in clinical decision-making, further long-term clinical verification is needed to confirm them.

## Data Availability

The original contributions presented in the study are included in the article/**Supplementary Material**. Further inquiries can be directed to the corresponding authors.
