# Supplementary figures and images for: Construction of a prognostic survival model for colorectal cancer patients using CT image texture analysis: a prospective cohort study
Source: Front Oncol. 2026 Jan 12;15:1738696. doi: 10.3389/fonc.2025.1738696 (PMC12832443; doi:10.3389/fonc.2025.1738696)

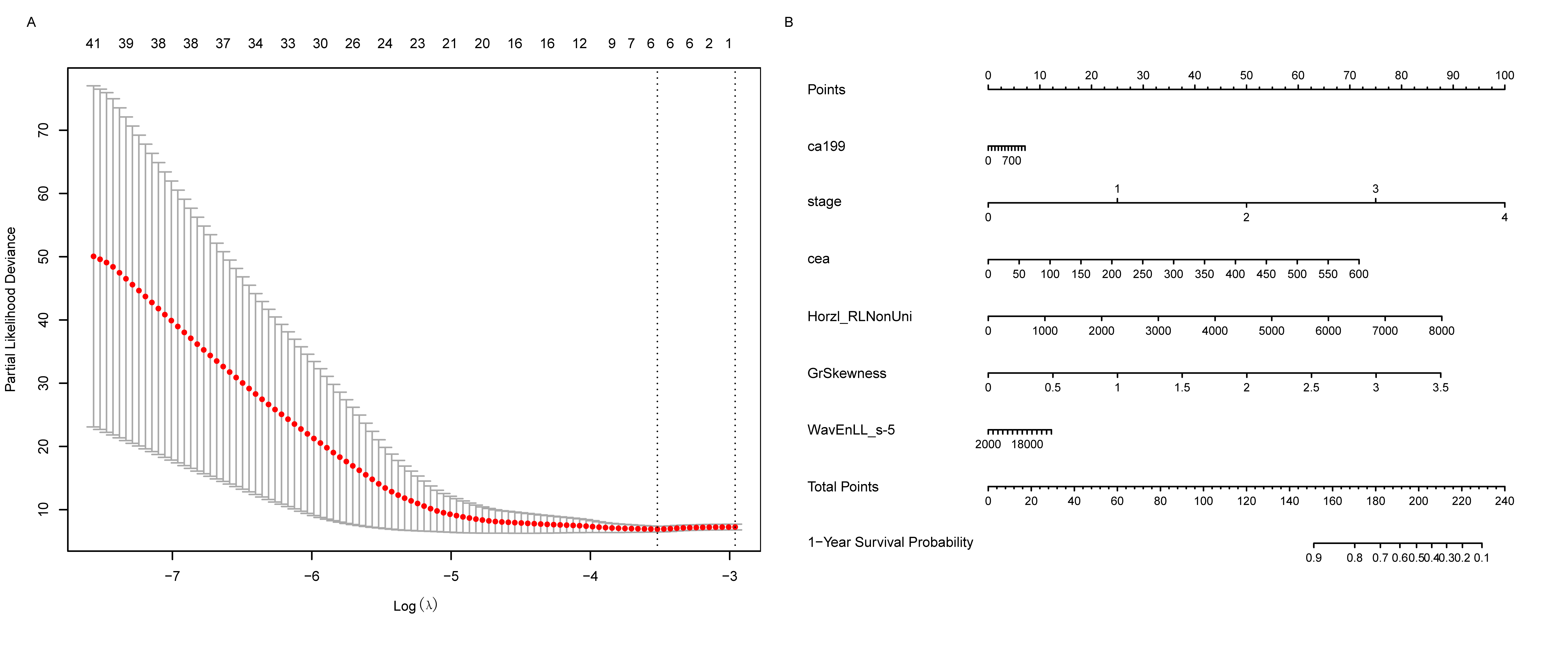

Supplement: Supplementary file 3 [file Image1.tif]

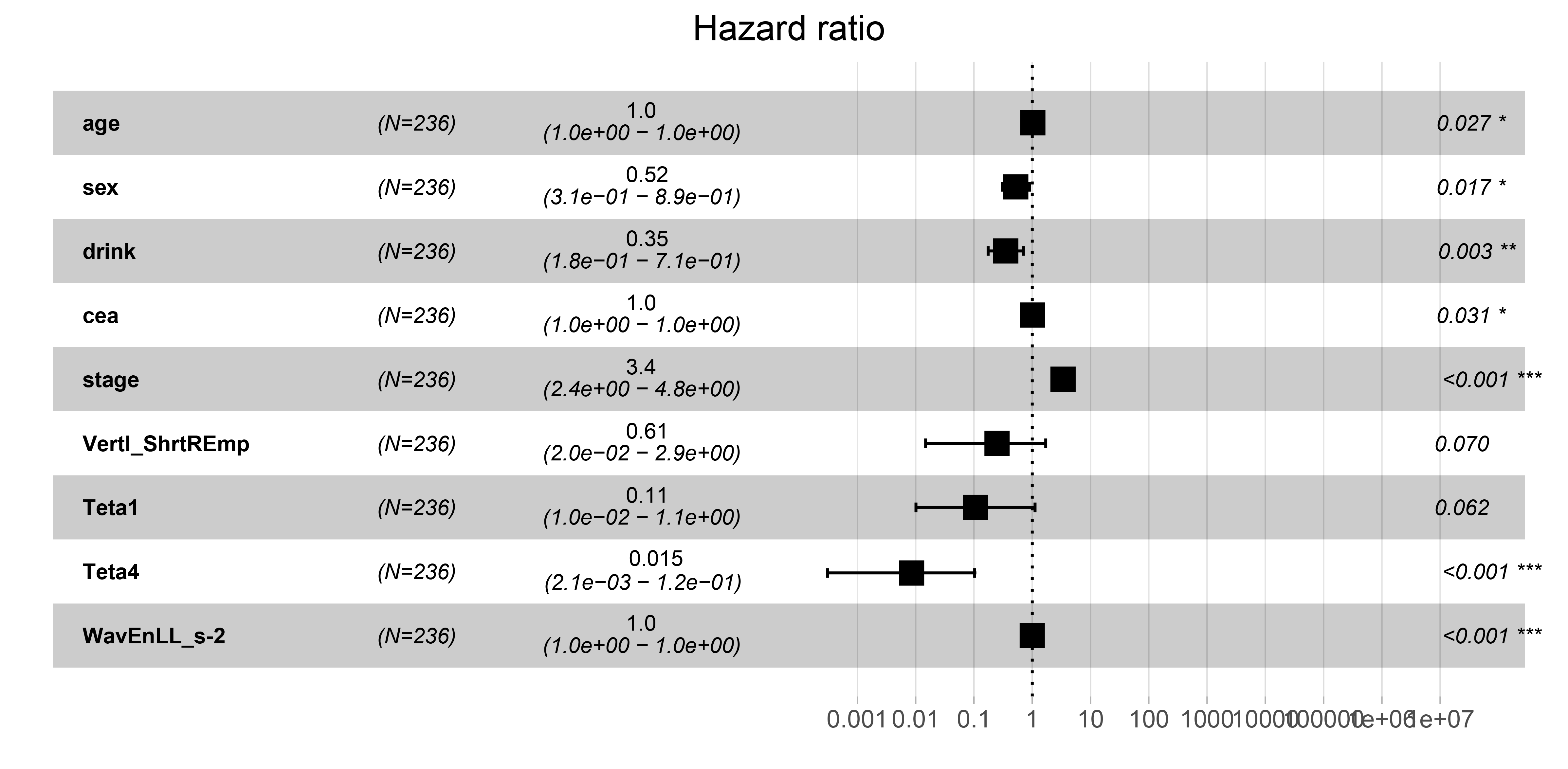

Supplement: Supplementary file 4 [file Image2.tif]
